# First-principles calculations on dislocations in MgO

**DOI:** 10.1080/14686996.2024.2393567

**Published:** 2024-08-19

**Authors:** Shin Kiyohara, Tomohito Tsuru, Yu Kumagai

**Affiliations:** a Institute for Materials Research, Tohoku University, Sendai, Japan; b Nuclear Science and Engineering Center, Japan Atomic Energy Agency, Ibaraki, Japan

**Keywords:** Dislocation, first-principles calculation, ceramics

## Abstract

While ceramic materials are widely used in our society, their understanding of the plasticity is not fully understood. MgO is one of the prototypical ceramics, extensively investigated experimentally and theoretically. However, there is still controversy over whether edge or screw dislocations glide more easily. In this study, we directly model the atomic structures of the dislocation cores in MgO based on the first-principles calculations and estimate the Peierls stresses. Our results reveal that the screw dislocation on the primary slip system exhibits a smaller Peierls stress than the edge dislocation. The tendency is not consistent with metals, but rather with TiN, suggesting a characteristic inherent to rock-salt type materials.

## IMPACT STATEMENT

Performing highly accurate computational methods – specifically, a combination of direct atomic modeling and first-principles calculations – to estimate the Peierls stresses of MgO.

## GRAPHICAL ABSTRACT

**Figure:**
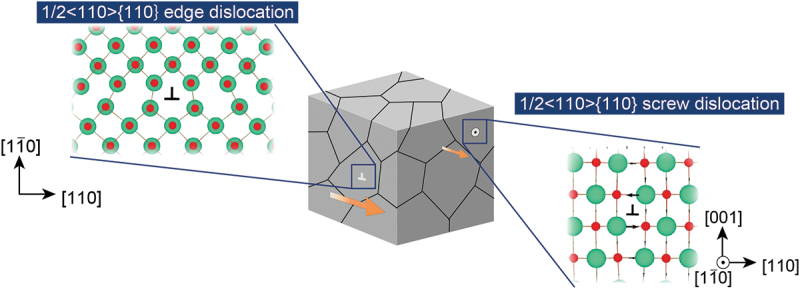

## 1. Introduction

Ceramic materials have a wide variety of functions and are indispensable in our society. Despite the versatility of their properties, their inherent brittleness limits their applications. For several decades, including recent years, much effort has been devoted developing ceramics with plasticity [1–6], e.g. by introducing highly dense defects, controlling microstructures, and utilizing external fields. Ceramics with high plasticity are easily processed and withstand harsh environments, leading to a wider range of industrial applications.

Dislocations are one of the lattice defects in crystalline materials, playing a dominant role in material plasticity [7]. When crystalline materials deform, the movement of dislocations results in smaller stresses than when the grains themselves move. Peierls stress is defined as the maximum stress required for a dislocation to move to an adjacent stable position at 0 K [8,9]. It can be measured experimentally; however, reported values for the same material often show large deviations due to unintentional point defects in the test specimens or differences in experimental conditions [10–15]. On the other hand, theoretical simulation is a suitable tool for estimating true Peierls stresses because it is basically calculated at 0 K and examines pristine materials [16–18].

There are two main computational approaches to calculate Peierls stresses and dislocation core structures. One is the Peierls–Nabarro (PN) model [8,9,19], which is still actively used despite its half-century history. In the model, the dislocation energies are divided into two contributions. The first is the elastic energy described by the elastic theory, while the second is the generalized stacking fault energies (GSFE), which are estimated using interatomic potentials or first-principles calculations [20–25]. The PN model has the advantage of less computational costs, but it cannot fully incorporate atomic relaxation during dislocation glide, which may have a potential error in the Peierls stresses. An alternative approach which can overcome the disadvantage of the PN model is the direct modelling of the dislocation core structures [26–37]. In principle, increasing the size of the supercells should systematically improve the accuracy, yet it also drastically increases computational costs. Hereafter, we refer to the direct modelling approach of the core structures as an atomistic simulation, as in Ref. [36], to distinguish it from the PN model approach.

In metals, dislocation core structures have been studied for several decades with atomistic simulations based on first-principles calculations. In particular, screw dislocations of body-centered-cubic (BCC) metals have been intensively investigated [26–30] because they have extraordinarily high Peierls stresses [38] and do not follow the Schmid law [39]. Furthermore, the effects of doping around the dislocation cores on dislocation glide have also been investigated [32–34]. However, there are limited studies on estimating the Peierls stresses of ceramics using atomistic simulations based on first-principles calculations, with the exception of TiN and Si [35–37].

MgO has a simple crystalline structure, making it one of the most typical examples of ceramics, and thus its mechanical properties have been intensively investigated experimentally [40–45] and theoretically [46–48].

Previous studies on the Peierls stresses in MgO are summarized in Table 1. They elucidated that the primary slip system of MgO at low temperature is 1/2<110>{110} [40], but it is still controversial over whether edge or screw dislocations glide more easily. Experimentally, the extrapolated critical resolved shear stress (CRSS) at 0 K, namely the Peierls stress, of MgO is approximately 150 MPa [12]. However, Gorum et al. experimentally measured that the CRSS of MgO at low contamination was about 40 MPa and that at high contamination was about 180 MPa [41], which means point defects largely effect on Peierls stresses. The Peierls stresses of the 1/2<110>{110} edge and screw dislocations can be separately estimated using transmission electron microscopy (TEM); Singh et al. estimated 60MPa for the edge and 170 MPa for the screw dislocation [42]. On the other hand, Moriyoshi et al. concluded that the Peierls stresses of both the edge and screw dislocations are about 70 MPa from the in-situ TEM observations [43].

**Table 1.** Previous reports on the calculated and experimental Peierls stresses for the four dislocations in MgO considered in this study. Our calculated values are also tabulated. Those marked with a single asterisk were not identified whether they were from the edge or screw. Values for TiN, derived from atomic models using first-principles calculations [36], are also listed.

Type	Previous reports (MPa)		This study (MPa)	Previous theoretical values for TiN [36]
	Experiment	Theoretical simulation		
Edge {100}	–	1160 [46], 300 ± 100 [48]	219	8700-9600
Edge {110}	150* [12], 60 [42], 70 [43], 40* [41]	20 [46], 80 ± 30 [48]	122	1300-1400
Screw {100}	–	1530 [46], 1600 ± 220 [47]	7533	>15300
Screw {110}	150* [12], 170 [42], 70 [43], 40* [41]	40 [46], 150 ± 70 [47]	61	400-700

Theoretically, Amanda et al. analyzed the temperature dependence of the screw dislocation by using the PN model and dislocation dynamics simulations [47,48]. The results show that (i) the simulated temperature-behaviour based on the double kink mechanism was consistent with experiments, (ii) the Peierls stress of the screw dislocation (~150 MPa), was larger than that of the edge dislocation (~80 MPa), and (iii) the screw dislocation was dominant for the plastic deformation like BCC metals. These are consistent with the experimental CRSS [12] (Table 1) and the fact that the screw dislocations in the TEM images are longer than the edge dislocations [49]. Still, the values are inconsistent with those of the low contamination sample and in-situ TEM observation [41,43], which may be due to the limit of the phenomenological model.

Therefore, in this study, we model dislocations in MgO by atomistic simulation and estimate their Peierls stresses from first principles. Consequently, we have found that the Peierls stress for the 1/2<110>{110} screw dislocation is about half of that for its edge dislocation counterpart.

## 2. Computational procedures

### 2.1. Dislocation models and energies

**Figure 1.**
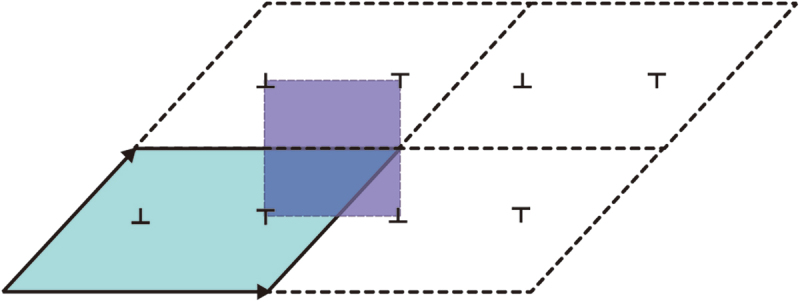
Schematic showing the dislocation dipole structure used in this study. The area shaded in light green represents a supercell, and the dotted areas show the repeated cells. The area shaded in dark blue marks a quadrupole structure.

To retain periodic boundary conditions, we need to introduce two equivalent dislocations with opposite Burgers vectors, namely a dislocation dipole, into the supercell. Figure 1 shows a schematic of our model containing a dislocation dipole. Considering translation symmetry, it is noted that the dipoles form dislocation quadrupoles (dark blue shaded area in Figure 1), whose arrangements reduce dislocation interactions [50]. In this study, the supercell lattice vectors were determined to ensure that the quadrupoles nearly form squares, with the difference between the two sides being less than 1 Å.

Using the elastic theory under periodic boundary conditions [51], we calculated the atomic displacements introduced by the elastic field generated by the dislocation quadrupole. In addition, to cancel out the strain introduced by the insertion of the quadrupole, we applied a homogeneous strain to the simulation cell which is given by

$$\varepsilon_{\mathrm{ij}}=\frac{b_{i}A_{j}+b_{j}A_{i}}{2S},$$

where $b_{i}$ and $A_{i}$ are components of the Burgers vector and dipole cut vector, respectively, and S is the area of the supercell perpendicular to the dislocation line.

The dislocation energies including the elastic energies depend on the supercell sizes. Therefore, we calculated the dislocation core energies as follows:

$$E_{\mathrm{core}}=\left(E_{\mathrm{tot},\;\mathrm{dipole}}-E_{\mathrm{tot},\;\mathrm{bulk}}-E_{\mathrm{elas}}\right)/2l,$$

where $E_{\mathrm{tot},\;\mathrm{dipole}}$ and $E_{\mathrm{tot},\;\mathrm{bulk}}$ represent the total energies of the supercell with the dislocation dipole and the bulk supercell, respectively. The ‘2’ in the denominator represents the number of dislocations per supercell and l is the length of the dislocation line. $E_{\mathrm{elas}}$ is the elastic energy and decomposed as follows:

$$E_{\mathrm{elas}}=E_{\mathrm{dipole}}+E_{\mathrm{dipole}-\mathrm{img}},$$

where $E_{\mathrm{dipole}}$ is a dislocation dipole interaction in the supercell and $E_{\mathrm{dipole}-\mathrm{img}}$ is that between periodic images. In this study, $E_{\mathrm{dipole}}$ and $E_{\mathrm{dipole}-\mathrm{img}}$ are estimated using the calculated elastic tensor and the anisotropic elastic theory [51,52], which is implemented in the babel program [53]. $E_{\mathrm{elas}}$ depends on the core radius of dislocations, which is set to 2*b*, where *b* is the magnitude of the Burgers vector.

### 2.2. Computational details

First-principles calculations were performed using the projector augmented-wave (PAW) method [54] as implemented in the Vienna ab initio simulation package (VASP) [55,56]. The Perdew–Burke–Ernzerhof functional tuned for solids (PBEsol) [57] was used for exchange – correlation functional. PAW data sets with radial cutoffs of 1.06 and 0.80 Å for Mg and O, respectively, were employed. Mg 3*s* and O 2*s* and 2*p* orbitals were considered as valence electrons. Internal atomic positions were optimized with fixed lattice constants until the residual forces on the atoms were reduced to less than 0.005 eV/Å. The plane-wave cutoff energy was set to 400 eV for the optimization.

After structure optimization, we estimated the Peierls stresses and potentials using the nudged elastic band (NEB) method [39]. When one dislocation moves, the quadrupole structure in Figure 1 deforms, which modifies the elastic energies in the supercell [27,51]. To avoid this, we concurrently moved both dislocations in the same direction. We set the number of transition images as four. The transition states were optimized until the residual forces converged to less than 0.03 eV/Å.

## 3. Results

Elastic properties play a dominant role in dislocation energy and dynamics, especially in the long-range regime. Therefore, we initially confirmed that the PBEsol functional calculations are in excellent agreement with the experimental values [58] as shown in Table 2.

**Table 2.** Calculated elastic constants in GPa.

	*C*_11_	*C*_12_	*C*_44_
This study	301	92	146
Experiments [58]	298	95	156

We then calculated the GSFE on the {100} and {110} planes (see Supplementary Figure S1). The predicted glide direction with the minimum energy is 1/2<110> for both planes, which is consistent with the minimum Burgers vector in MgO. Furthermore, the unstable energies are 1.96 J/m^2^ for the {100} plane and 1.08 J/m^2^ for the {110} plane, resulting in that the primary slip system is 1/2 < 110>{110} system.

### 3.1. Edge dislocations

**Figure 2.**
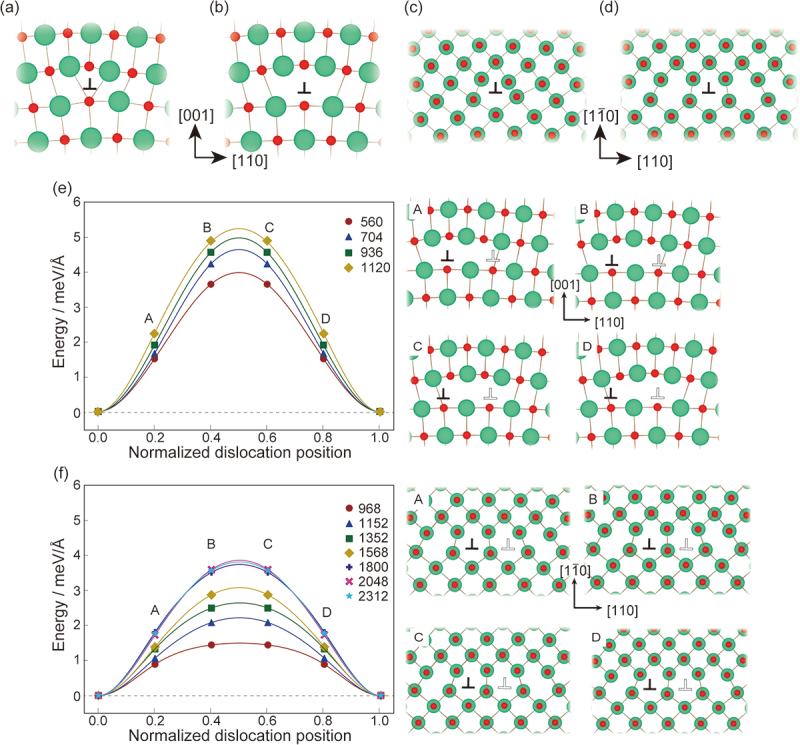
Atomic core structures and energy profiles during glide of the edge dislocations. Core structures of the (a, b) 1/2<110>{100} and (c, d) 1/2<110>{110} (a, c) before and (b, d) after relaxation. The green and red circles are Mg and O atoms, respectively. Bonds with a length 1.15 times longer than Mg-O (2.10 Å) in the bulk are illustrated throughout this paper. (e, f) energy profiles of the 1/2<110>{100} and 1/2<110>{110} edge dislocations, respectively. The horizonal dotted lines represents zero energy. The initial and final structures are set at 0 and 1 in the *x*-axis, respectively. The numbers in the figures show the numbers of the atoms in the supercells. Black and white dislocation labels (‘⊥’) are initial and final positions of the dislocation centres. Letters of ‘A’ to ‘D’ in the panels of the energy profiles represent the transition states, whose core structures are illustrated in the right figures.

Figures 2(a,b) show the atomic structures of the 1/2<110>{100} edge dislocation before and after relaxation, respectively. The atomic structures of the entire supercell are shown in the Supplementary Figure S2. The atoms outside the dislocation cores are only slightly displaced by the elastic field generated by the dislocations. On the other hand, the atoms optimized in the vicinity of the dislocation cores are relatively significantly displaced, up to 0.37 Å, by the structure optimization. This indicates the difficulty of elasticity theory in accurately describing the atomic structures of the dislocation cores. Both Mg and O atoms near the cores are found to be undercoordinated. The density of states (DOS) of the supercells with and without the dislocation shown in the Supplementary Figure S3(a,b) is found to be close to that of the unit cell. However, the dislocation core generates a small tail at the valence band top, as indicated by the black arrow in the figure. Thus, the edge dislocation can modify the electronic and optical properties.

The atomic structures before and after relaxation of the 1/2<110>{110} edge dislocation are shown in Figures 2(c,d), respectively. Compared to the 1/2<110>{100} edge dislocation, its undercoordinated atoms are widely distributed on the (1$\overset{ˉ}{1}$0) slip plane. This extended core structures have been observed experimentally by the scanning TEM [59]. It is also noted that the atomic structure of the 1/2<110>{110} edge dislocation agrees well with that in TiN [36], whereas that of the 1/2<110>{100} edge dislocation in MgO differs from that in TiN [36] (see also Supplementary Figure S4). In TiN, we can clearly see extra two half planes in the 1/2<110>{100} edge dislocation, but we cannot see them in MgO due to the extension on the (001) plane of the dislocation. Their GSFE profiles in the <100> direction on {100} surface are shown in Supplementary Figure S5. Although the shapes of the profiles are similar to each other, TiN has higher GSFE than MgO, which may reduce the distribution of the core structure in TiN. Thus, the atomic structures of the same dislocation in the same rock-salt structure depend on, e.g. the elastic tensor and the nature of the chemical bonds.

We then calculated the Peierls stresses using NEB. The energies of the initial and final structures should be identical because the final structures are merely translated from the initial one by the Burgers vector. Indeed, the energy differences are found to be approximately less than 1 meV/Å at most (see Supplementary Figure S6), which attributes to the numerical error caused by the discrete Fourier transform. To reduce the errors, we symmetrize the profile by flipping it and taking the average of the profile before and after flipping it. The raw transition energy profiles are shown in Supplementary Figure S6.

The results are shown in Figures 2(e,f), Table 1, and Supplementary Table S1. The energy profiles and corresponding quantities are found to converge with a sufficiently large number of atoms in the simulation cells for the later discussion, especially about the order of the Peierls stresses. For both the 1/2<110>{100} and 1/2<110>{110} edge dislocations, it is found that the atoms moved only about 0.5 Å at most during dislocation glide; nevertheless, the dislocation positions were moved by 1/2[110], namely 2.97 Å. The 1/2<110>{110} edge dislocation has a smaller Peierls stress than the 1/2<110>{100} edge dislocation, which agrees with the experimental and theoretical finding that the main slip system of MgO is 1/2<110>{110} [40,46,48].

**Figure 3.**
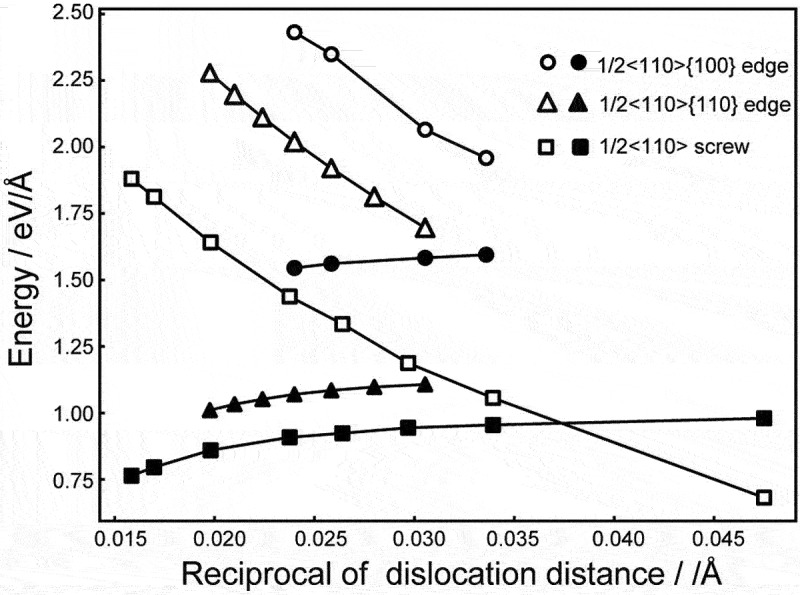
Dislocation elastic and core energies as a function of the distance between dislocation dipole. Filled marks are dislocation core energies while open marks are elastic energies.

Figure 3 shows the elastic and core energies of the dislocations. The elastic energy of the 1/2<110>{110} edge dislocation differs from that of the 1/2<110>{100} edge dislocation. They should be the same in an isotopic medium. Thus, the energy difference is due to the elastic anisotropy. Also, the core energy of the 1/2<110>{110} edge dislocation is smaller than that of the 1/2<110>{100} edge dislocation, which is consistent with the fact that the main slip system is 1/2<110>{110} in MgO. It is noted that the core energies do not converge with respect to the supercell size in our calculations. One potential cause is electrostatic interactions between effective charges at the dislocation cores under periodic boundary conditions [60]. Such effective charges, however, do not affect the Peierls stresses in this study because the relative positions of the core structures under periodic boundary conditions remain constant during the dislocation glide.

### 3.2. Screw dislocations

**Figure 4.**
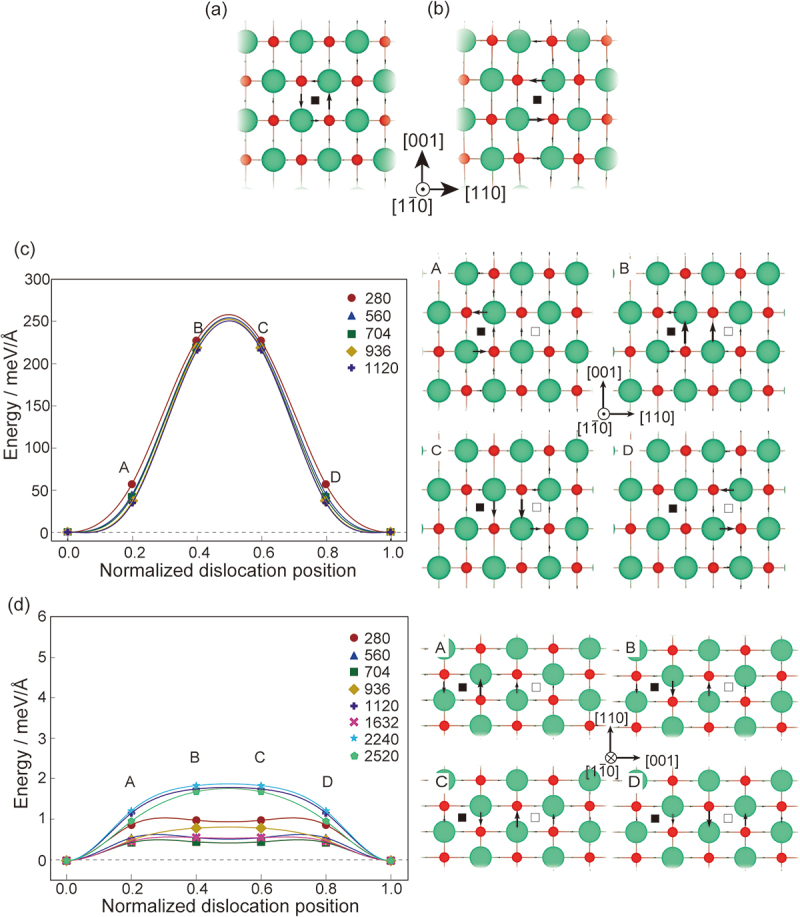
Atomic core structures and energy profiles during glide of the screw dislocations. (a, b) core structures (a) before and (b) after relaxation. (c, d) energy profiles of the 1/2<110>{100} and 1/2<110>{110} screw dislocations, respectively. Black arrows are differential displacement maps (see text for details). The length of the arrows is normalized the largest differential displacement. Black and white squares are initial and final positions of the dislocation centres, respectively. The other details are the same as in Figure 2.

Figures 4(a,b) show atomic displacements of the 1/2<110> screw dislocations before and after relaxation, where the atomic displacements to [1$\overset{ˉ}{1}$0] direction are plotted by the differential displacement (DD) method [61], where the lengths of the arrows are proportional to the relative shift of two neighboring atoms along the [1$\overset{ˉ}{1}$0] direction (vertical direction of the paper) when inserting the dislocation in the bulk. The DD maps are overlapped on atomic structure of the bulk in Figure 4(a) or the optimized structures in Figure 4(b). The initial structure before relaxation in Figure 4(a) has a circuit core structure, while the optimized structure in Figure 4(b) has an core structure mainly on (110) plane. Furthermore, atoms in the vicinity of the dislocation core have displacements perpendicular to the dislocation line, namely the [110] direction, which is similar to the 1/2<111> screw dislocation of iron [62].

We then glided the dislocation positions on the (001) and (110) planes to estimate their Peierls stresses (see Figures 4(c,d), Table 1, and Supplementary Table S1). Interestingly, the two slip systems share the same atomic structures, but the Peierls stress in the {100} plane is more than a hundred times than that in the {110} plane. We reveal that the large difference is originated from the DD plot of the dislocation. Originally, the dislocation is distributed on the (110) plane as shown in Figure 4(b). For glide on the (001), the dislocation is distributed on the (001) plane during the glide (see the right panel labeled B in Figure 4(c)) and redistributed on the adjacent (110) plane (see the right panel labeled D in Figure 4(c)). On the other hand, for glide on the (110) plane, the dislocation distribution is kept on the (110) plane during the glide, which results in the small Peierls stress.

### 3.3. Comparison with the previous simulations and experiments

In the theoretical studies shown in Table 1, it was generally concluded that (i) the primary slip system is 1/2<110>{110} and (ii) the edge dislocations exhibit smaller Peierls stresses than the screw dislocations for both slip systems. While our calculation results agree with the conclusion (i), the Peierls stress of the edge dislocation is higher than that of the screw dislocation for the {110} plane. The discrepancy should result from the non-elastic energy in the PN mode estimated by the GSFE, which incorporates atomic relaxation only perpendicular to the slip plane. The core structure of the 1/2<110>{110} edge dislocation in Figure 2(f) was relaxed to the [110] and [1$\overset{ˉ}{1}$0] directions. In such cases, the relaxation cannot be adequately described by the GSFE. For the 1/2<110>{110} screw dislocation, we can see the displacements perpendicular to the dislocation line in Figure 4(b). On the other hand, such displacements are not seen in the previous report [47]. It is noteworthy that the magnitude relationship between the edge and screw dislocations aligns well with that observed in TiN, which was estimated using the direct modelling and first-principles calculations, similar to our study.

The TEM observations concluded that, for the primary slip system, the screw dislocations have a Peierls stress approximately three times higher than or equal to that of the edge dislocations [42,43], which is inconsistent with our calculations. In mechanical tests, it is well known that inevitable point defects significantly affect Peierls stresses, which may result in the inconsistency. Another possible cause is a Peach–Koehler force [7], which is an interaction between dislocations. Moreover, the calculated small Peierls stress of the screw dislocation and its compact core structure in Figure 4(d) may suggest that the dislocation multiplication is originated from the double-cross slip mechanism [43].

## 4. Conclusions

In this study, we modeled the atomic structures of edge and screw dislocation cores for the 1/2<110>{100} and {110} slip systems using first-principles calculations. For the edge dislocations, the 1/2<110>{100} dislocation exhibits a slightly higher Peierls stress compared to the 1/2<110>{110} dislocation. For the screw dislocations, the 1/2<110>{100} dislocation demonstrates a significantly higher Peierls stress than the 1/2<110>{110} dislocation. This notable difference in the screw dislocations is attributed to the spatial distribution of their cores. Additionally, within the 1/2<110>{110} slip system, the screw dislocation presents a lower Peierls stress than the edge dislocation. In metals, screw dislocations exhibit Peierls stresses several times higher than those of edge dislocations, while TiN shows a similar behavior to MgO. Hence, the relationship between edge and screw dislocations in the primary slip system could be a distinctive characteristic found in the ceramics with the rock-salt structure.

## Supplementary Material

Supplemental Material

Supplemental Material

## References

[1] Nieh TG, Wadsworth J, Wakai F. Recent advances in superplastic ceramics and ceramic composites. Int Mater Rev. 1991;36:146–9.

[2] Chen H, Wei T, Zhao K, et al. Room‐temperature plastic inorganic semiconductors for flexible and deformable electronics. InfoMat. 2021;3(1):22–35. doi: 10.1002/inf2.12149

[3] Li J, Cho J, Ding J, et al. Nanoscale stacking fault–assisted room temperature plasticity in flash-sintered TiO 2. Sci Adv. 2019;5(9):1–9. doi: 10.1126/sciadv.aaw5519 PMC6984969 32047855

[4] Shen C, Li J, Niu T, et al. Achieving room temperature plasticity in brittle ceramics through elevated temperature preloading. Sci Adv. 2024;10(16):1–10. doi: 10.1126/sciadv.adj4079 PMC11023556 38630827

[5] Oshima Y, Nakamura A, Matsunaga K. Extraordinary plasticity of an inorganic semiconductor in darkness. Science (80-). 2018;360(6390):772–774. doi: 10.1126/science.aar6035 29773747

[6] Porz L, Klomp AJ, Fang X, et al. Dislocation-toughened ceramics. Mater Horiz. 2021;8(5):1528–1537. doi: 10.1039/D0MH02033H 34846461

[7] Anderson PM, Hirth JP, Lothe J. Theory of dislocations. United Kingdom: Cambridge University Press; 2017.

[8] Peierls R. The size of a dislocation. Proc Phys Soc. 1940;52(1):34–37. doi: 10.1088/0959-5309/52/1/305

[9] Nabarro FRN. Dislocations in a simple cubic lattice. Proc Phys Soc. 1947;59(2):256–272. doi: 10.1088/0959-5309/59/2/309

[10] Cottrell AH, Bilby BA. Dislocation theory of yielding and strain ageing of iron. Proc Phys Soc Sect A. 1949;62(1):49–62. doi: 10.1088/0370-1298/62/1/308

[11] Caillard D. An in situ study of hardening and softening of iron by carbon interstitials. Acta Mater. 2011;59(12):4974–4989. doi: 10.1016/j.actamat.2011.04.048

[12] Amodeo J, Merkel S, Tromas C, et al. Dislocations and plastic deformation in MgO crystals: a review. Crystals. 2018;8(6):240–253. doi: 10.3390/cryst8060240

[13] Monnet G, Pouchon MA. Determination of the critical resolved shear stress and the friction stress in austenitic stainless steels by compression of pillars extracted from single grains. Mater Lett. 2013;98:128–130. doi: 10.1016/j.matlet.2013.01.118

[14] Noradila AL, Sajuri Z, Syarif J, et al. Effect of strain rates on tensile and work hardening properties for Al-zn magnesium alloys. IOP Conf Ser Mater Sci Eng. 2013;46:0–6. doi: 10.1088/1757-899X/46/1/012031

[15] Bednarczyk W, Wątroba M, Jain M, et al. Determination of critical resolved shear stresses associated with slips in pure Zn and Zn-ag alloys via micro-pillar compression. Mater Des. 2023;229:111897.

[16] Bulatov V, Cai W. Computer simulations of dislocations. United Kingdom: Oxford University Press; 2006.

[17] Tsuru T. Descriptions of dislocation via first principles calculations the plaston concept. Singapore: Springer Nature Singapore; 2022. p. 91–115.

[18] Clouet E. Ab initio models of dislocations. In: W. Andreoni, S. Yip, editors. Handbook of materials modeling: methods: theory and modeling. Switzerland, Cham: Springer International Publishing; 2020. p. 1503–1524.

[19] Eshelby JD. LXXXII. Edge dislocations in anisotropic materials. Lond, Edinburgh, Dublin Philos Mag J Sci. 1949;40(308):903–912. doi: 10.1080/14786444908561420

[20] Zimmerman A, Gao H, Abraham FF. Generalized stacking fault energies for embedded atom FCC metals. Modell Simul Mater Sci Eng. 2000;8(2):103–115. doi: 10.1088/0965-0393/8/2/302

[21] Ogata S, Li J, Yip S. Ideal pure shear strength of aluminum and copper. Science (80-). 2002;298(5594):807–811. doi: 10.1126/science.1076652 12399585

[22] Zhang SH, Beyerlein IJ, Legut D, et al. First-principles investigation of strain effects on the stacking fault energies, dislocation core structure, and Peierls stress of magnesium and its alloys. Phys Rev B. 2017;95(22):1–12. doi: 10.1103/PhysRevB.95.224106

[23] Liu X, Pei Z, Eisenbach M. Dislocation core structures and Peierls stresses of the high-entropy alloy NiCoFeCrMn and its subsystems. Mater Des. 2019;180:107955. doi: 10.1016/j.matdes.2019.107955

[24] Ferré D, Carrez P, Cordier P. Modeling dislocation cores in SrTi O3 using the Peierls-Nabarro model. Phys Rev B - Condens Matter Mater Phys. 2008;77(1):1–7. doi: 10.1103/PhysRevB.77.014106

[25] Li Y, Liu X, Zhang P, et al. Theoretical insights into the peierls plasticity in SrTiO3 ceramics via dislocation remodelling. Nat Commun. 2022;13(1):1–10. doi: 10.1038/s41467-022-34741-4 36376322 PMC9663548

[26] Ventelon L, Willaime F. Core structure and Peierls potential of screw dislocations in α-Fe from first principles: cluster versus dipole approaches. J Comput Mater Des. 2007;14(S1):85–94. doi: 10.1007/s10820-007-9064-y

[27] Clouet E, Ventelon L, Willaime F. Dislocation core energies and core fields from first principles. Phys Rev Lett. 2009;102(5):1–4. doi: 10.1103/PhysRevLett.102.055502 19257518

[28] Itakura M, Kaburaki H, Yamaguchi M. First-principles study on the mobility of screw dislocations in bcc iron. Acta Mater. 2012;60(9):3698–3710. doi: 10.1016/j.actamat.2012.03.033

[29] Dezerald L, Rodney D, Clouet E, et al. Plastic anisotropy and dislocation trajectory in BCC metals. Nat Commun. 2016;7(1):11695. doi: 10.1038/ncomms11695 27221965 PMC4894953

[30] Kraych A, Clouet E, Dezerald L, et al. Non-glide effects and dislocation core fields in BCC metals. Npj Comput Mater. 2019;5(1):1–8. doi: 10.1038/s41524-019-0247-3

[31] Clouet E, Caillard D, Chaari N, et al. Dislocation locking versus easy glide in titanium and zirconium. Nat Mater. 2015;14(9):931–936. doi: 10.1038/nmat4340 26147845

[32] Romaner L, Ambrosch-Draxl C, Pippan R. Effect of rhenium on the dislocation core structure in tungsten. Phys Rev Lett. 2010;104(19):195503. doi: 10.1103/PhysRevLett.104.195503 20866976

[33] Tsuru T, Chrzan DC. Effect of solute atoms on dislocation motion in Mg: an electronic structure perspective. Sci Rep. 2015;5:8793. 25740411 10.1038/srep08793 PMC4350096

[34] Tsuru T, Suzudo T. First-principles calculations of interaction between solutes and dislocations in tungsten. Nucl Mater Energy. 2018;16:221–225. doi: 10.1016/j.nme.2018.07.007

[35] Pizzagalli L, Beauchamp P, Jónsson H. Calculations of dislocation mobility using nudged elastic band method and first principles DFT calculations. Philos Mag. 2008;88(1):91–100. doi: 10.1080/14786430701767402

[36] Yadav SK, Ramprasad R, Misra A, et al. Core structure and Peierls stress of edge and screw dislocations in TiN: a density functional theory study. Acta Mater. 2014;74:268–277. doi: 10.1016/j.actamat.2014.04.047

[37] Salamania J, Sangiovanni DG, Kraych A, et al. Elucidating dislocation core structures in titanium nitride through high-resolution imaging and atomistic simulations. Mater Des. 2022;224:111327. doi: 10.1016/j.matdes.2022.111327

[38] Vítek V. Thermally activated motion of screw dislocations in B.C.C metals. Phys Status solidi. 1966;18(2):687–701. doi: 10.1002/pssb.19660180221

[39] Duesbery MS, Vitek V. Plastic anisotropy in b.c.c. transition metals. Acta Mater. 1998;46(5):1481–1492. doi: 10.1016/S1359-6454(97)00367-4

[40] Hulse CO, Copley SM, Pask JA. Effect of crystal orientation on plastic deformation of magnesium oxide. J Am Ceram Soc. 1963;46(7):317–323. doi: 10.1111/j.1151-2916.1963.tb11738.x

[41] Gorum AE, Luhman WJ, Pask JA. Effect of impurities and heat‐treatment on ductility of MgO. J Am Ceram Soc. 1960;43(5):241–246. doi: 10.1111/j.1151-2916.1960.tb14591.x

[42] Singh RN, Coble RL. Dynamic dislocation behavior in “‘pure’” magnesium oxide single crystals. J Appl Phys. 1974;45(3):981–989. doi: 10.1063/1.1663445

[43] Moriyoshi Y, Kingrey WD, Vander Sande JB. Dislocation motion in magnesium oxide. J Mater Sci. 1978;13:2507–2510.

[44] Issa I, Amodeo J, Réthoré J, et al. In situ investigation of MgO nanocube deformation at room temperature. Acta Mater. 2015;86:295–304. doi: 10.1016/j.actamat.2014.12.001

[45] Bhowmik A, Lee J, Britton TB, et al. Deformation behaviour of [001] oriented MgO using combined in-situ nano-indentation and micro-Laue diffraction. Acta Mater. 2018;145:516–531. doi: 10.1016/j.actamat.2017.12.002

[46] Carrez P, Ferré D, Cordier P. Peierls–Nabarro modelling of dislocations in MgO from ambient pressure to 100 GPa. Model Simul Mater Sci Eng. 2009;17(3):035010. doi: 10.1088/0965-0393/17/3/035010

[47] Amodeo J, Carrez P, Devincre B, et al. Multiscale modelling of MgO plasticity. Acta Mater. 2011;59(6):2291–2301. doi: 10.1016/j.actamat.2010.12.020

[48] Amodeo J, Carrez P, Cordier P. Modelling the effect of pressure on the critical shear stress of MgO single crystals. Philos Mag. 2012;92(12):1523–1541. doi: 10.1080/14786435.2011.652689

[49] Appel F, Bethge H, Messerschmidt U. Dislocation motion and multiplication at the deformation of MgO single crystals in the high voltage electron microscope. Phys Status Solidi. 1977;42(1):61–71. doi: 10.1002/pssa.2210420104

[50] Bigger JRK, McInnes DA, Sutton AP, et al. Atomic and electronic structures of the 90° partial dislocation in silicon. Phys Rev Lett. 1992;69(15):2224–2227. doi: 10.1103/PhysRevLett.69.2224 10046430

[51] Cai W, Bulatob VV, Chang J, et al. Periodic image effects in dislocation modelling. Philos Mag. 2003;83(5):539–567. doi: 10.1080/0141861021000051109

[52] Clouet E. Elastic energy of a straight dislocation and contribution from core tractions. Philos Mag. 2009;89(19):1565–1584. doi: 10.1080/14786430902976794

[53] Clouet E babel. Available from: http://emmanuel.clouet.free.fr/Programs/Babel/

[54] Blöchl PE. Projector augmented-wave method. Phys Rev B. 1994;50(24):17953–17979. doi: 10.1103/PhysRevB.50.17953 9976227

[55] Kresse G, Furthmüller J. Efficient iterative schemes for ab initio total-energy calculations using a plane-wave basis set. Phys Rev B. 1996;54(16):11169–11186. doi: 10.1103/PhysRevB.54.11169 9984901

[56] Kresse G, Joubert D. From ultrasoft pseudopotentials to the projector augmented-wave method. Phys Rev B. 1999;59(3):1758–1775. doi: 10.1103/PhysRevB.59.1758

[57] Perdew JP, Ruzsinszky A, Csonka GI, et al. Restoring the density-gradient expansion for exchange in solids and surfaces. Phys Rev Lett. 2008;100(13):136406. doi: 10.1103/PhysRevLett.100.136406 18517979

[58] Yoneda A. Pressure derivatives of elastic constants of single crystal MgO and MgAl2O4. J Phys Earth. 1990;38(1):19–55. doi: 10.4294/jpe1952.38.19

[59] Wang Z, Saito M, McKenna KP, et al. Polymorphism of dislocation core structures at the atomic scale. Nat Commun. 2014;5(1):3239. doi: 10.1038/ncomms4239 24476810

[60] Ukita M, Nakamura A, Yokoi T, et al. Electronic and atomic structures of edge and screw dislocations in rock salt structured ionic crystals. Philos Mag. 2018;98(24):2189–2204. doi: 10.1080/14786435.2018.1478146

[61] Vítek V, Perrin RC, Bowen DK. The core structure of ½(111) screw dislocations in b.c.c. crystals. Philos Mag. 1970;21(173):1049–1073. doi: 10.1080/14786437008238490

[62] Clouet E, Ventelon L, Willaime F. Dislocation core field.Ii. Screw dislocation in iron. Phys Rev B. 2011;84(22):224107. doi: 10.1103/PhysRevB.84.224107 19257518

