# Supplementary figures and images for: First-principles calculations on dislocations in MgO
Source: Sci Technol Adv Mater. 2024 Aug 19;25(1):2393567. doi: 10.1080/14686996.2024.2393567 (PMC11370692; doi:10.1080/14686996.2024.2393567)

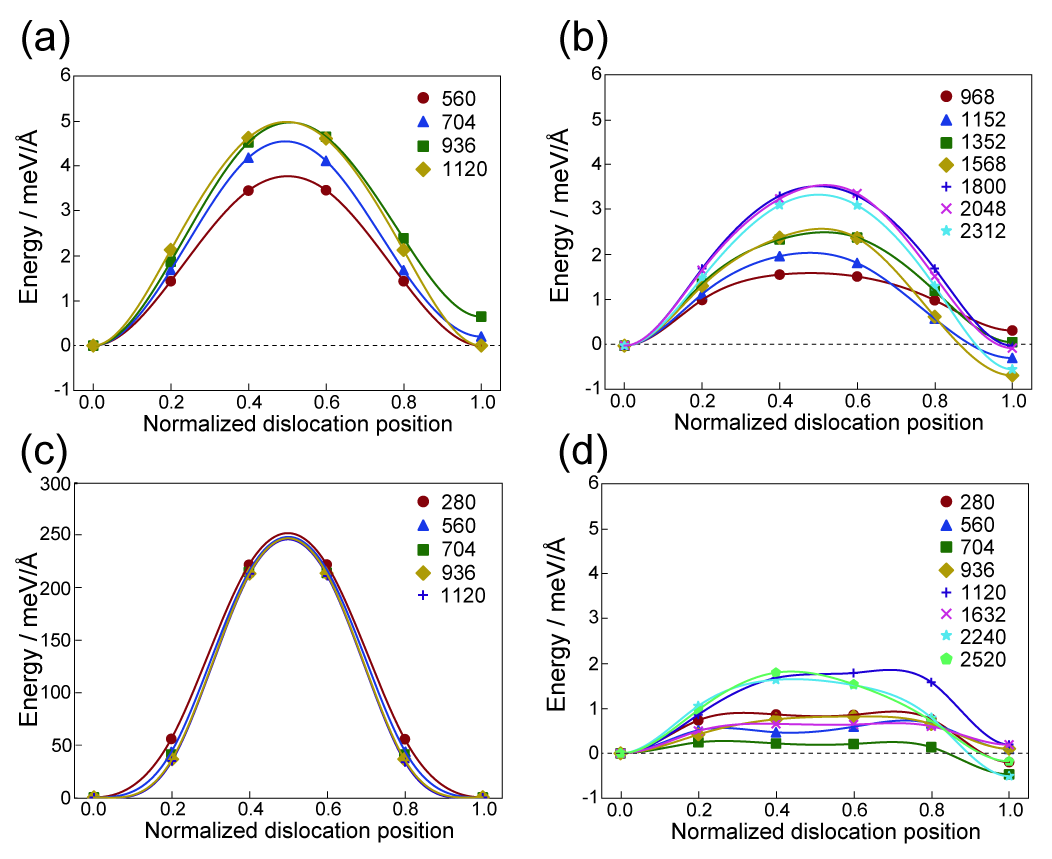

Supplement: Supplemental Material [file TSTA_A_2393567_SM3808.tif]
