# Supplementary material for: First-principles calculations on dislocations in MgO
Source: Sci Technol Adv Mater. 2024 Aug 19;25(1):2393567. doi: 10.1080/14686996.2024.2393567 (PMC11370692; doi:10.1080/14686996.2024.2393567)
Supplement: Supplemental Material [file TSTA_A_2393567_SM3806.docx]

**First-principles Calculations on Dislocations in MgO**

Shin Kiyohara^a,*^, Tomohito Tsuru^b^, Yu Kumagai^a^

^a^Institute for Materials Research, Tohoku University, 2-1-1 Katahira, Aoba-ku, Sendai, 980-8577, Japan; ^b^Nuclear Science and Engineering Center, Japan Atomic Energy Agency, 2-4 Shirakata, Tokai-mura, Ibaraki 319-1195, Japan

Generalized stacking fault energy:

The generalized stacking fault energy (GSFE) is a energy difference between two adjacent planes during shear deformation in a specific slip direction on a slip plane. We translated a part of grains on the {100} and {110} planes in the supercell systematically, optimized the perpendicular coordinates of the atoms to the slip planes, and estimated the energy difference between the deformed supercells and the bulk. The schematic is shown in Fig. S1 (a).


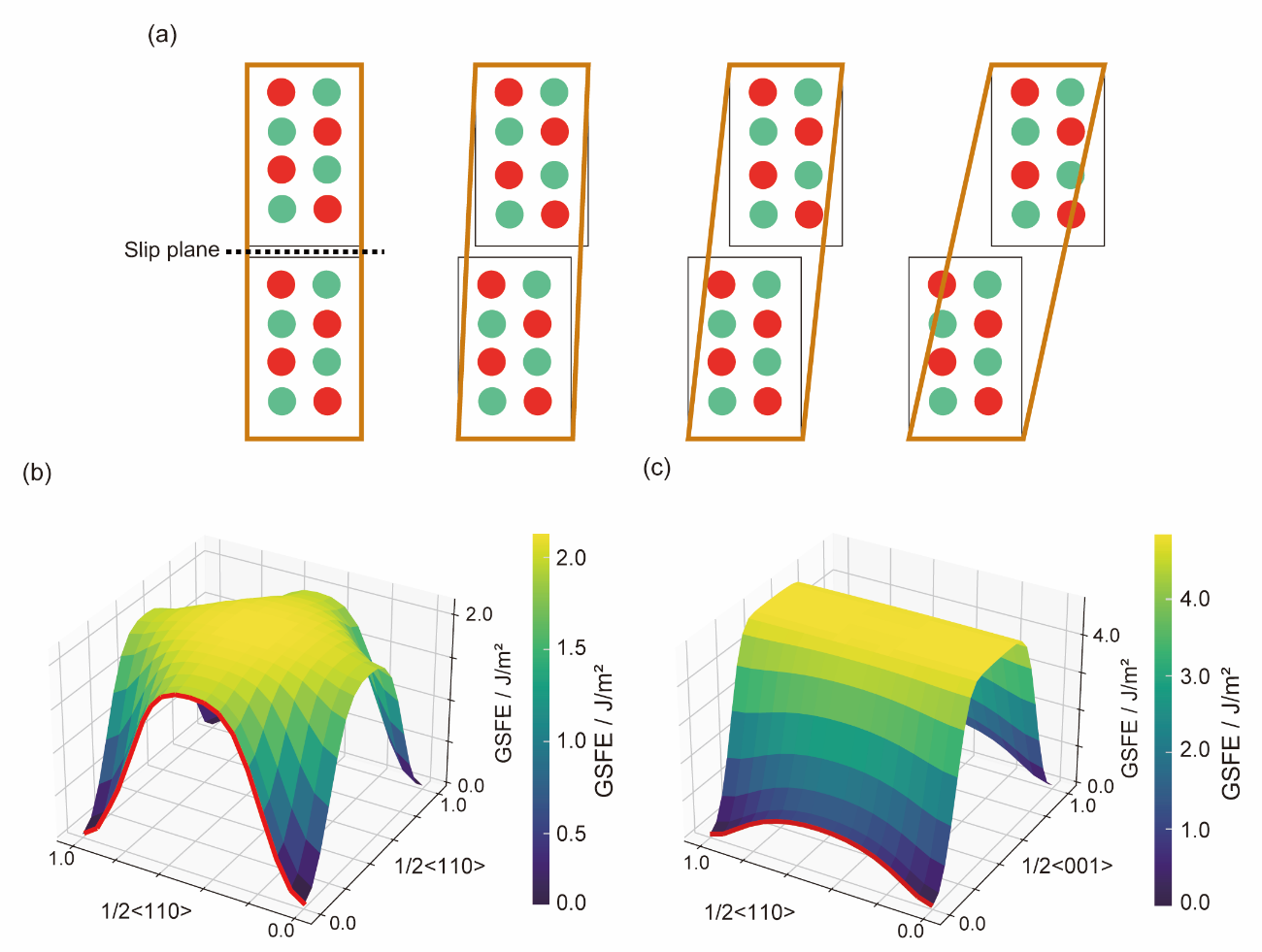


Figure S1. Generalized stacking fault energy. (a) Schematic of the supercells for calculating the GSFEs. The black rectangles surrounding the green and red circles represent the grains adjacent to the slip plane. The orange parallelograms represent the lattice of the supercells. (b, c) Calculated GSFE for the {100} and {110} surfaces, respectively. The red lines show the minimum energy paths. The unstable energies on the paths are 1.96 J/m^2^ for the {100} and 1.08 J/m^2^ for the {110}, respectively.


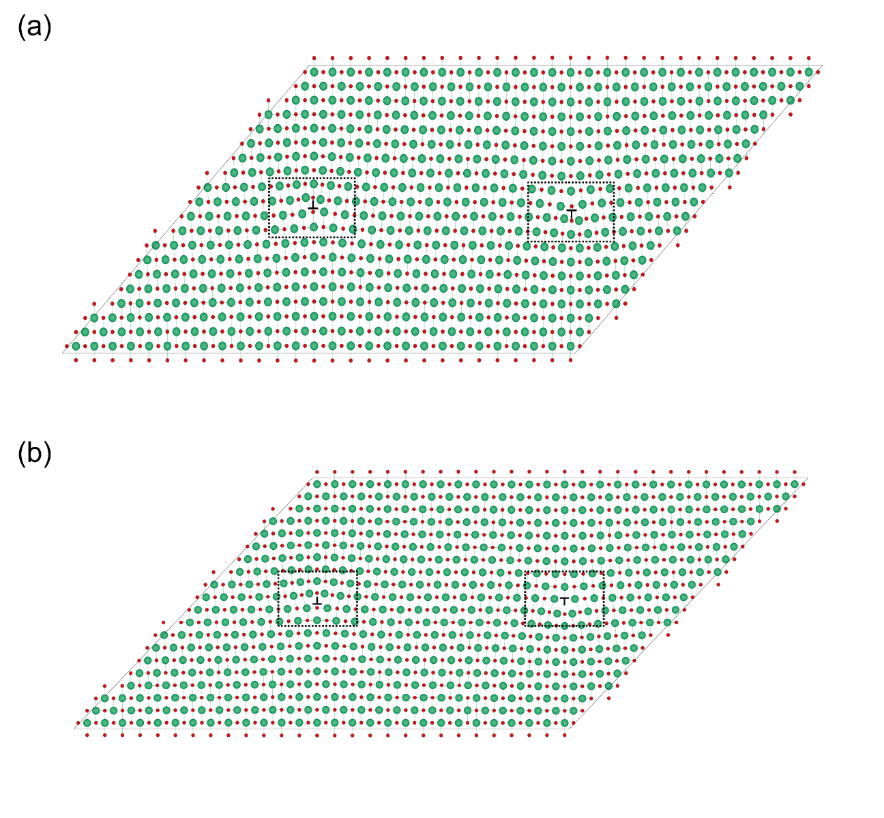


Figure S2. Atomic structures of the 1/2<110>{100} edge dislocation. (a) Before and (b) after relaxation. The green and red circles are Mg and O atoms, respectively. Bonds with a length within 1.15 times that of the bulk (2.10 Å) are illustrated.


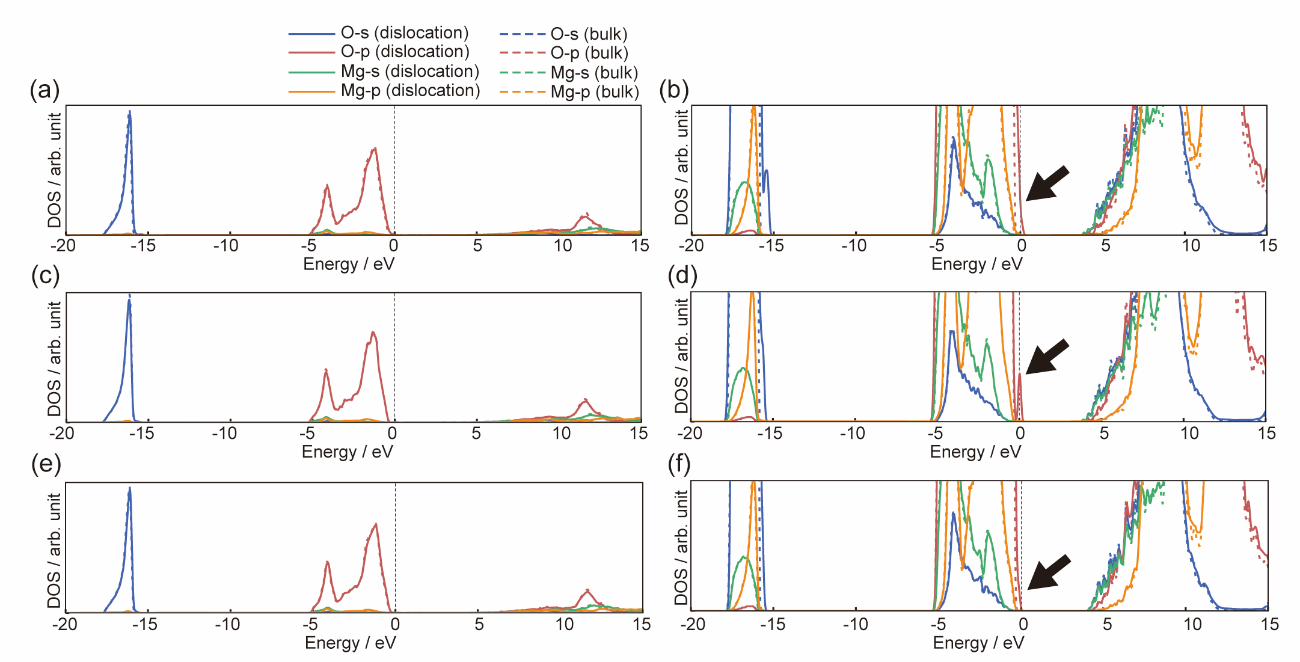


Figure S3. The density of states (DOS) of the (a, b) 1/2<110>{100} edge dislocation, (c, d) 1/2<110>{110} edge dislocation, and (e, f) 1/2<110>{100} and {110} screw dislocation models. Figures of (b, d, f) are magnified by 80 of (a, c, e) figures, respectively. Solid and dotted lines represent the DOS of the dislocations and bulk, respectively. Black arrows in (b, d, f) represent the states generated by the dislocations.


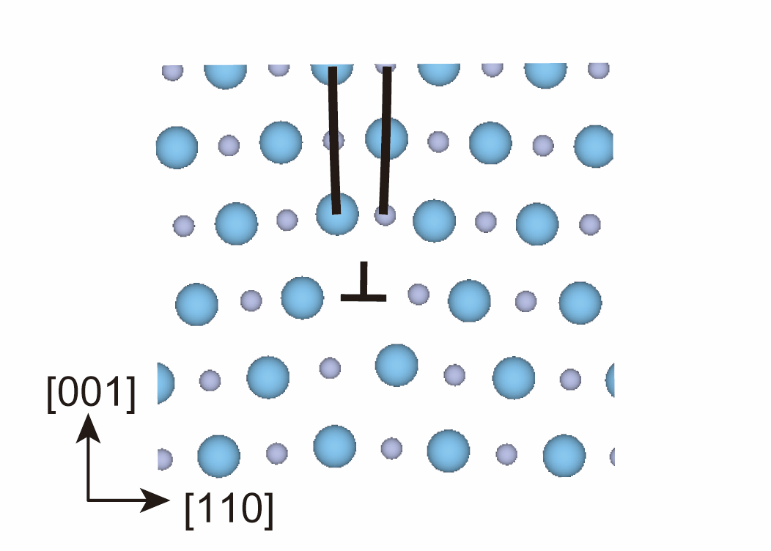


Figure S4. Atomic core structures of the 1/2<110>{100} edge dislocation of TiN. Blue and gray circles are Ti and N, respectively. Black bold lines represent inserted planes.

The atomic structure was calculated following the same procedure as TiN. The simulation cell contained 936 atoms. PAW data sets with radial cutoffs 1.5 Å of Ti and 0.79 Å and N were employed. 3d and 4s orbitals for Ti and 2s and 2p orbitals for N were considered as valence electrons. The plane-wave cutoff energy was set to 400 eV for the structural optimization.


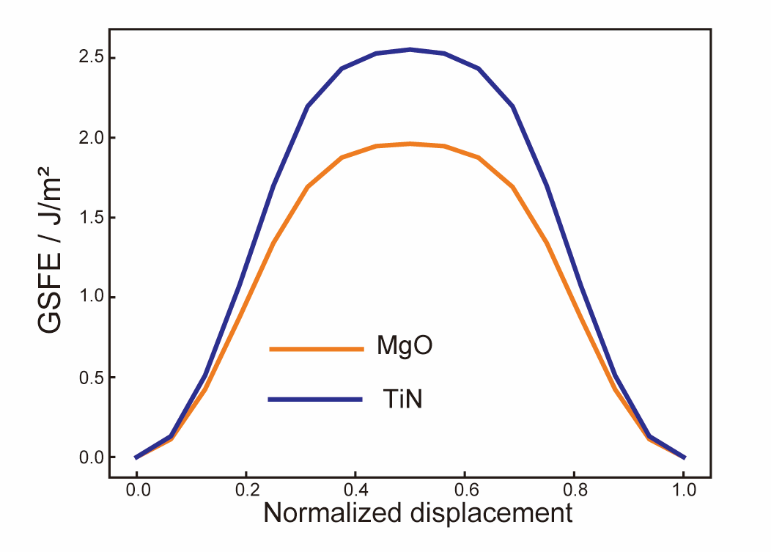


Figure S5. GSFE profiles of <100> direction on {100} surface of MgO and TiN. Orange and blue lines are MgO and TiN, respectively.

**
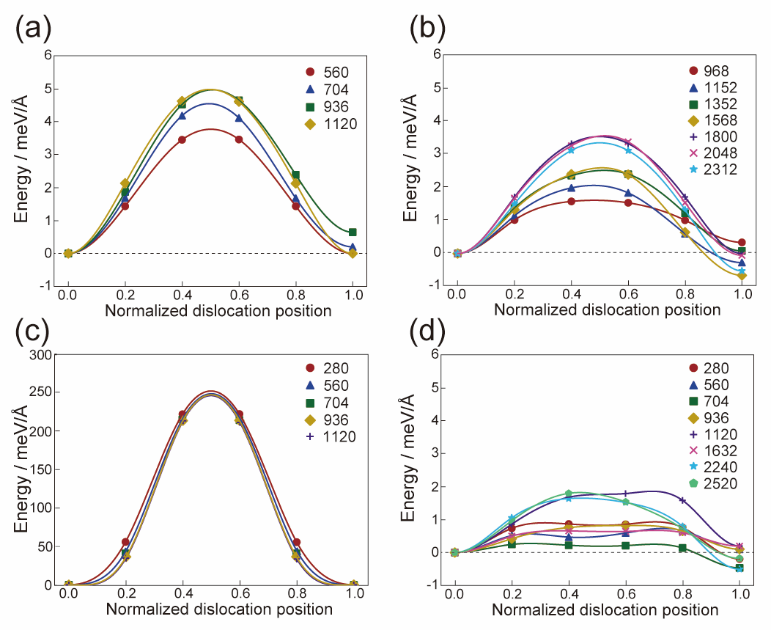
**

Figure S6. The energy profiles during dislocation glide. (a) 1/2<110>{100} edge dislocation; (b) 1/2<110>{110} edge dislocation; (c) 1/2<110>{100} screw dislocation; (d) 1/2<110>{110} screw dislocation. The horizonal dotted lines represents 0 meV/Å.

Table S1. The calculated Peierls stresses and energies. Some of the Peierls energies are not listed on the table because they have semi-stable points on the profiles due to their small cell size.

| Type | Number of atoms | Peierls stress (MPa) / energy (meV/Å) |
| --- | --- | --- |
| Edge {100} | 560  704  936  1120 | 180 / 3.2  206 / 3.4  209 / 4/0  219 / 4.2 |
| Edge {110} | 968  1152  1352  1568  1800  2048  2312 | 68 / 0.8  81 /1.3  97 / 1.5  102 / 1.7  127 / 2.1  123 / 2.2  122 / 2.2 |
| Screw {100} | 280  560  704  936  1120 | 7727 / 226  7653 / 223  7628 / 221  7634 / 222  7533 / 220 |
| Screw {110} | 280  560  704  936  1120  1632  2240  2520 | 68 / -  40 / -  30 / -  34 / -  77 / 1.5  30 / -  77 / 1.5  61 / 1.4 |
